# Feasibility, Acceptability, and Efficacy of Home-Based Transcranial Direct Current Stimulation on Pain in Older Adults with Alzheimer’s Disease and Related Dementias: A Randomized Sham-Controlled Pilot Clinical Trial

**DOI:** 10.3390/jcm12020401

**Published:** 2023-01-04

**Authors:** Geraldine Martorella, Hongyu Miao, Duo Wang, Lindsey Park, Kenneth Mathis, JuYoung Park, Julia Sheffler, Lisa Granville, Antonio L. Teixeira, Paul E. Schulz, Hyochol Ahn

**Affiliations:** 1 College of Nursing, Florida State University, Tallahassee, FL 32306, USA; 2 Department of Statistics, Florida State University, Tallahassee, FL 32306, USA; 3 The University of Texas Health Science Center at Houston, McGovern Medical School, Houston, TX 77030, USA; 4 Phyllis & Harvey Sandler School of Social Work, Florida Atlantic University College of Social Work and Criminal Justice, Boca Raton, FL 33431, USA; 5 College of Medicine, Florida State University, Tallahassee, FL 32306, USA

**Keywords:** transcranial direct current stimulation, Alzheimer’s disease and related dementias, pain, efficacy, acceptability, feasibility

## Abstract

Although transcranial direct current stimulation (tDCS) is emerging as a convenient pain relief modality for several chronic pain conditions, its feasibility, acceptability, and preliminary efficacy on pain in patients with Alzheimer’s disease and related dementias (ADRD) have not been investigated. The purpose of this pilot study was to assess the feasibility, acceptability, and preliminary efficacy of 5, 20-min home-based tDCS sessions on chronic pain in older adults with ADRD. We randomly assigned 40 participants to active (*n* = 20) or sham (*n* = 20) tDCS. Clinical pain intensity was assessed using a numeric rating scale (NRS) with patients and a proxy measure (MOBID-2) with caregivers. We observed significant reductions of pain intensity for patients in the active tDCS group as reflected by both pain measures (NRS: Cohen’s d = 0.69, *p*-value = 0.02); MOBID-2: Cohen’s d = 1.12, *p*-value = 0.001). Moreover, we found home-based tDCS was feasible and acceptable intervention approach for pain in ADRD. These findings suggest the need for large-scale randomized controlled studies with larger samples and extended versions of tDCS to relieve chronic pain on the long-term for individuals with ADRD.

## 1. Introduction

Alzheimer’s disease and related dementias (ADRD) affects more than 5.5 million individuals in the United States [1] and this number is projected to triple in the next 30 years [2,3]. Approximately 60% of persons with ADRD experience pain [4,5] and at least 45% experience chronic pain [6]. Chronic pain is the second most reported reason for decreased quality of life in persons with ADRD [7] and remains undertreated [8]. Our group [9,10] and others [11,12,13,14] showed pain in ADRD is associated with behavioral and psychological symptoms of dementia (BPSD) as well as functional impairment. Despite the lack of specific guidelines for this population, the prescription of analgesic medications remains a mainstay, which often produce significant adverse effects and safety issues [8,15]. Given the increasing evidence on safety and efficacy of several nonpharmacological interventions for pain, these interventions should be prioritized.

Recent evidence suggests that alterations in pain-related brain mechanisms may contribute to various chronic pain conditions [16,17,18,19,20]. Indeed, because ADRD involves brain changes long before the diagnosis, it has been suggested that chronic pain is associated with dementia underlying pathophysiology [21,22]. Experimental studies have shown that reaction to pain of individuals with ADRD is intensified [23,24,25,26,27,28], and that neurodegeneration and brain structural changes affect descending pain inhibition and may explain this phenomenon in this population [29]. Therefore, there is heightened interest in nonpharmacological interventions targeting pain-related brain function in this population. One promising treatment is transcranial direct current stimulation (tDCS) with the anode over the primary motor cortex (M1) and the cathode over the contralateral supraorbital (SO) area (M1-SO applied tDCS), as it can change brain activity in a noninvasive, painless, and safe way [30,31,32]. tDCS is categorized by the U.S. Food and Drug Administration as a “non-significant risk” device [33,34].

Our group [35,36,37,38,39] and others [40,41,42,43,44,45] have shown that clinical-based tDCS intervention improves pain in older adults by directly modulating activity in brain areas involved in pain processing. In addition, tDCS has been shown to improve memory and general cognitive function in people with ADRD [46]. Moreover, conventionally offered in a clinic setting, we have tested the feasibility, acceptability, and efficacy of tDCS in the home setting with older adults and obtained statistically and clinically significant results on pain [47,48]. This approach, enhanced by recent technological advances [49], offers convenience and accessibility to evidence-based nonpharmacological treatment of pain for older adults, including persons with ADRD who have difficulties attending clinic-based sessions [46,50,51,52,53]. In fact, home-based tDCS has been safely used to improve cognitive or memory problems among persons with ADRD [54,55]. However, no research to date has examined whether home-based, remotely supervised tDCS can reduce pain in persons with ADRD.

Thus, the purpose of this pilot study was to assess the feasibility, acceptability, and preliminary efficacy of this novel nonpharmacological treatment option in persons with ADRD in planning for a larger scale study. We examined home-based, remotely supervised, M1-SO applied tDCS in 40 persons with early-stage ADRD using an experimenter- and participant-blinded, randomized, sham-controlled, parallel group (1:1 for two groups) pilot clinical trial. Stimulation parameters were chosen based on evidence-based guidelines and prior tDCS research [30,31,35,45,49,56].

## 2. Materials and Methods

### 2.1. Design

The protocol has been registered at www.clinicaltrials.gov, accessed on 29 November 2022 (NCT04457973). After obtaining ethical approval from the Florida State University’s Institutional Review Board (STUDY00002524), we conducted a double-blind, randomized, sham-controlled, phase II parallel-group pilot study with two groups (sham and active tDCS). In our previous study [35], we observed high retention rate (>95%) and the effect size of active tDCS (Cohen’s d) on clinical pain compared to sham tDCS was 1.0. With the sample size of 20 per group, we planned to detect the expected effect size of 1.0 with 80% power at a significance level of 0.025. Forty community-dwelling participants with early-stage ADRD were randomly assigned at a ratio of 1:1 to receive home-based active tDCS (*n* = 20) or sham tDCS (*n* = 20) using the order of entrance in the study and a randomization list generated via SAS software (version 9.4) by a statistician with no clinical involvement in this trial. The randomization balanced the allocation of with respect to distributions of age, race, sex, and dementia severity.

### 2.2. Participants

Individuals were 50 to 90 years old and considered eligible if they (1) had early-stage ADRD, (2) had caregiver-reported chronic pain (average pain in the past 3 months ≥ 3 out of 10 on a NRS from 0 to 10), (3) had a caregiver willing to participate in the study who sees the participant at least 10 hours/week, (4) could speak and read English, and (5) had no plans to change medication regimens during the trial. The diagnosis of early-stage ADRD was verified by the study physician and the following inclusion criteria were used: Clinical Dementia Rating scores (generally from 0.5 to 1.0) [57], Mini-Mental Status Exam score (MMSE, generally from 16 to 23), or the blind/telephone version of the Montreal Cognitive Assessment (MoCA, generally from 16 to 26). Participants were excluded if they had any concurrent medical conditions that could confound the interpretation of outcome measures, pose a safety risk for any of the assessment or tDCS procedures, or preclude the successful completion of the protocol. Specific exclusion criteria were: (1) history of brain surgery, brain tumor, seizure, stroke, or intracranial metal implantation, (2) alcohol/substance use disorder, (3) severely diminished cognitive function (i.e., Mini-Mental Status Exam score ≤ 15), and (4) hospitalization within the preceding year for neuropsychiatric illness.

Individuals who were eligible and interested in participating were scheduled for a baseline visit with their caregiver, which took place 3 to 7 days prior to the tDCS intervention and included the following: acquisition of written informed consent; evaluation of clinical pain and pain-related cortical response; and training on home-based tDCS. Caregivers were also asked to give their informed consent, as their involvement was critical. Participants were then randomly assigned to active or sham tDCS. After the final treatment session in the 5-day intervention period, post-treatment measures were assessed, and the tDCS device was returned to the investigators. We also collected clinical pain data each month for three months after the completion of treatment to examine the maintenance of benefits.

### 2.3. tDCS and Sham Conditions

Based on our previous studies [35,39,47] and evidence-based guidelines for tDCS [30], home-based active tDCS with a constant current of 2 mA was applied for 20 minutes per session daily for 5 days via the Soterix 1 × 1 tDCS mini-CT Stimulator device with headgear and 5 × 7 cm saline-soaked surface sponge electrodes to maximize reproducibility and participant comfort. The anode was placed over primary motor cortex of the left hemisphere, and the cathode was placed over the right supraorbital area. The sponge electrodes snap into the custom headgear, which is secured to the participant’s head for simple and fail-safe electrode preparation [58]. Participants/caregivers administered a stimulation session via the Soterix 1 × 1 tDCS mini-CT Stimulator device only after being provided a single-use unlock code by the research staff, once proper contact quality is achieved (only the on/off button was adjustable by the study participants; they were not able to adjust the device settings). The tDCS device senses the contact between scalp and SNAPPad^®^ and displays whether the contact is poor, moderate, or good. At 20 minutes, the device turned off automatically, and study staff instructed the participant to remove the headset, discard sponges, and safely store all materials for the next session. For consistency and remote supervision, participants were instructed to sit in a comfortable chair and remain quiet while using the device at a predetermined time each weekday. For sham stimulation, the electrodes were placed in the identical positions as for active stimulation, but the stimulator delivered no electrical current except a 30-s ramp-up period at the beginning and end to mimic somatosensory perception of active tDCS. Electrodes can mitigate sensation to prevent subjects from guessing if they are receiving active or sham stimulation. This sham approached has been deemed reliable and indistinguishable from active tDCS [59,60].

### 2.4. Data Collection

All participants completed a thorough questionnaire to collect demographic and medical history details, including the use of medications. Outcome measures were taken at 4 time points (after treatment, months 1, 2, and 3 post-treatment). We maintained consistency by collecting all data at the same time of day for all participants. Of note, data were obtained from both patients and caregivers via paper questionnaires. For the posttreatment assessment, although, they were in the same room in our laboratory, they were not sitting next to each other and could not read each other’s responses to questionnaires. Follow-up assessments were done over the phone monthly for 3 months.

### 2.5. Clinical Pain Intensity

Clinical pain, our primary outcome, was measured via a numeric rating scale (NRS) based on recommendations from the Initiative on Methods, Measurement, and Pain Assessment in Clinical Trials (IMMPACT) for clinical trials involving chronic pain [61], with our primary endpoint at the end of the 5 treatment sessions and a secondary endpoint at 3 months. Clinical pain intensity was measured by asking participants to rate their average pain over the past 24 hours via NRS from 0 (no pain) to 100 (worst pain imaginable). Additionally, the Mobilization-Observation-Behavior-Intensity-Dementia (MOBID-2) scale [62,63], which includes 10 items (pain during specific activities/movement) and an overall pain intensity assessment using a NRS from 0 to 10, was used with caregivers. The MOBID-2 has a reported Cronbach’s alpha coefficient of ≥0.8 and is a well-validated measure with good ability to detect pain change in persons with ADRD [62,63]. Solely the NRS score (from 0 to 100) and the overall pain intensity score of the MOBID-2 (from 0 to 10) were used for the analysis.

### 2.6. Behavioral and Psychological Symptoms of Dementia (BPSD)

BPSD were measured with the help of caregivers using the Cohen-Mansfield Agitation Inventory (CMAI) long form [64] which comprises a 7-point Likert scale for frequency of 29 behaviors (e.g., verbal aggression, hitting, kicking, and pushing) that are summed to yield an overall score from 29 to 203. The CMAI has a reported Cronbach’s alpha coefficient of ≥0.7 and is a well-validated measure with good ability to detect BPSD change in persons with ADRD [65,66,67]. Complementarily, the Neuropsychiatric Inventory (NPI) [68,69] was also administered to evaluate neuropsychiatric symptomatology of dementia. The NPI examines the 4-point Likert scale for frequency and 3-point Likert scale for severity of 12 subdomains of dementia (e.g., delusions, hallucinations, agitation/aggression, dysphoria, anxiety, euphoria, apathy, disinhibition, irritability/lability, aberrant motor activity, night-time behavioral disturbances. and appetite and eating abnormalities) that are summed to yield an overall score from 0 to 144 (each item will be rated frequency X severity, totaling from 0 to 12), with higher scores indicating greater symptoms. Test–retest reliability was found to be 0.79 for frequency and 0.86 for sensitivity. Internal consistency has been reflected by a Cronbach’s alpha of 0.88 for the total NPI score. The NPI, a well-validated measure used in hundreds of trials, has been shown to be valid and reliable [70].

### 2.7. Feasibility

In accordance with CONSORT guidelines [71], we calculated the percentage of participants who (a) met the inclusion criteria, (b) agreed to be randomly assigned, (c) completed the full tDCS protocol, and (d) attended the follow-up assessment.

### 2.8. Acceptability

We collected data on the tDCS experience via a questionnaire, adapted from Gillick et al. [72] and Cha et al. [73], administrated to patients or caregivers (depending on who manipulated equipment) at the conclusion of tDCS treatment on a 0 (strongly disagree) to 10 (strongly agree) scale: (1) It was easy to prepare the device and accessories; (2) The device was unnecessarily complex; (3) The device was easy to use; (4) I felt the video conferences with a technical person were helpful; (5) I would imagine that most people would learn to use this device quickly; (6) The device was cumbersome to use; (7) I felt confident using the device; (8) I needed to learn a lot of things before I could get going with this device; (9) The effectiveness of the treatment increased over the course of treatment; and (10) Overall, I felt that transcranial electrical stimulation treatment benefited me. Participants/caregivers were also encouraged to elaborate on their answers in free form. In addition, we measured participant satisfaction with treatment using the Client Satisfaction Questionnaire (CSQ-8) [74,75]. The CSQ-8 comprises eight items that are summed to yield an overall score from 8 to 32, with higher scores indicating greater satisfaction. Moreover, we evaluated the presence and severity of possible side effects of treatment at the end of each session on a 0 (not at all) to 10 (highest degree) scale. The participants were asked in an open-ended manner whether they experienced any side effects, more specifically tingling, itching sensation, burning sensation, pain at the stimulation site, fatigue, nervousness, headache, difficulty concentrating, mood change, and changes in vision or visual perception. If any side effects were reported, the degree of relatedness to the intervention was assessed on a 5-point scale. This approach was used in our previous study and frequently in other studies, and no serious adverse events have been encountered [32,35,76].

### 2.9. Statistical Methods

All statistical analyses were performed with R Statistical Software (version 4.2.2; R Foundation for Statistical Computing, Vienna, Austria). Throughout the analyses, statistical significance was established on α = 0.05, and multiple testing correction was not performed since a pilot study is usually designed for hypothesis generation instead of hypothesis testing. Raw data were cleaned and then examined using the Shapiro test for normality assumption verification. Wilcoxon rank-sum tests were used for non-normally distributed outcomes (e.g., 5-day changes in NRS, CMAI, and NPI), and t-tests were employed for normally distributed outcomes (e.g., 5-day MOBID score changes). A multivariate linear regression model was then used together with backward stepwise model selection to examine certain potential confounding factors (i.e., gender, age, and race). To verify whether the efficacy of tDCS were sustained over 3 months after intervention, the NRS score changes as well as the MOBID score changes from baseline to month 3 after intervention were compared with those changes from baseline to Day 5 using Wilcoxon signed-rank test or paired *t*-test (where appropriate). Furthermore, profile analysis based on linear mixed-effects model was employed to verify whether the temporal trajectories of NRS changes and MOBID changes were the same between the sham and active groups, respectively. Moreover, to explore the relationships between the longitudinal NRS (or MOBID) changes (i.e., from baseline to Day 5, Month 1, Month 2, Month 3) and the potential confounding factors mentioned above, generalized estimating equation (GEE) model analysis was performed. Finally, we conducted sex-stratified analysis to explore the gender-specific outcome differences.

## 3. Results

**Figure 1:**
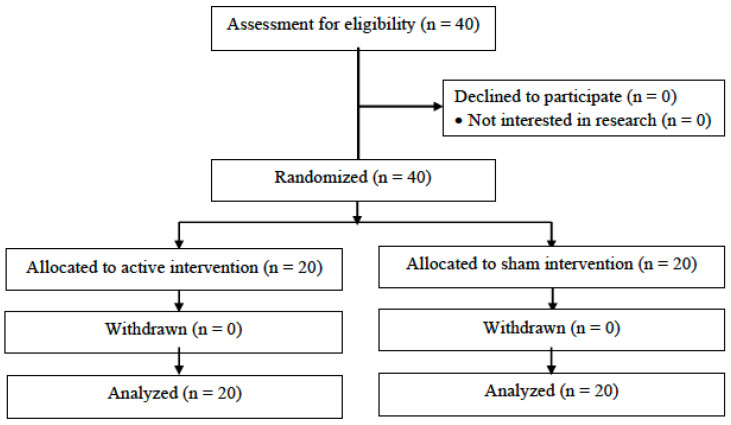
Study flow diagram.

A total of 40 participants were successfully recruited and randomly assigned to the active tDCS group (20 subjects) or the sham tDCS group (20 subjects) between September 2020 and October 2022. Figure 1 depicts the participants study flow (the detailed flow chart is available in Figure S1 in supplemental material). The mean age was 73 years (SD = 7.87), and most participants were white (90%) and women (72.5%). Table 1 presents detailed baseline demographic and clinical characteristics of the participants. No significant differences between groups were found at baseline for pain intensity (NRS and MOBID-2). No changes in medication regimens were reported during the trial.

### 3.1. Clinical Pain Intensity

The NRS changes from Day 0 to Day 5 were found significantly different between the two groups (Cohen’s d = 0.69, *p*-value = 0.02). The MOBID changes from baseline to Day 5 were found significantly different between the sham and active tDCS groups (Cohen’s d = 1.12, *p*-value < 0.01). Table 2 shows the NRS and MOBID score changes from baseline to Day 5 and to month 3 after intervention of the sham and the active tDCS groups. For NRS, the mean decrease was 13.55 ± 14.90 at Day 5 from baseline for the active group while it was only 3.30 ± 14.81 for the sham group; at 3-month after intervention, the mean decrease of NRS was 18.7 ± 27.48 for the active group while only 8.3 ± 27.59 for the sham group. The MOBID scores in average decreased by 2.70 ± 2.39 at day 5 from baseline for the active group while it was 0.40 ± 1.67 for the sham group; at 3-month after intervention, the MOBID scores decreased by 2.55 ± 3.46 for the active group and 0.30 ± 1.8 for the sham group, respectively.

Multivariate regression analysis was conducted to examine the effects of potential confounding factors like gender, age, and race on NRS and MOBID scores changes at day 5 from baseline, respectively (see Table S1 in supplemental material). The results showed that: (1) age was not significantly associated with either NRS or MOBID score changes; (2) both NRS and MOBID score changes were not significantly different between males and females; (3) race was not significantly associated with NRS and MOBID score changes. However, the different racial subgroups were insufficiently represented. Other potential confounding factors (such as history height, history weight and education) were also included in the multivariate linear model with stepwise model selection performed, and no significant confounders were detected.

**Figure 2:**
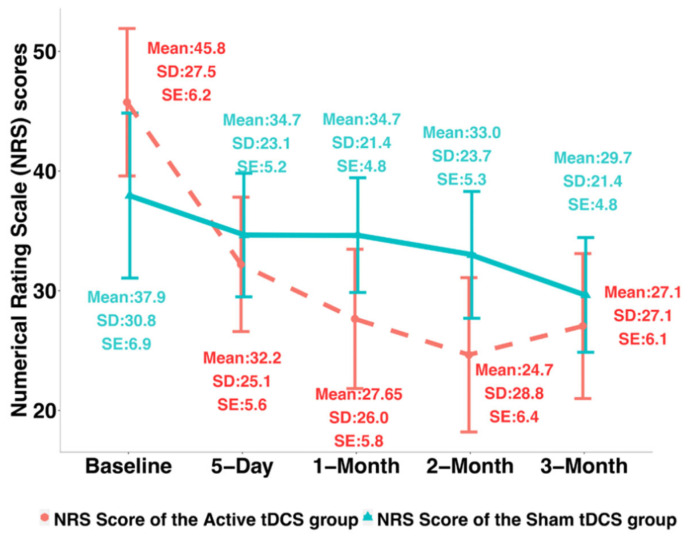
Numerical Rating Scale (NRS) scores over time of the active and the sham tDCS groups (*n* = 40). The dashed red line represents the mean NRS scores of the active tDCS group, and the solid green line represents the mean NRS score of the sham tDCS group.

**Figure 3:**
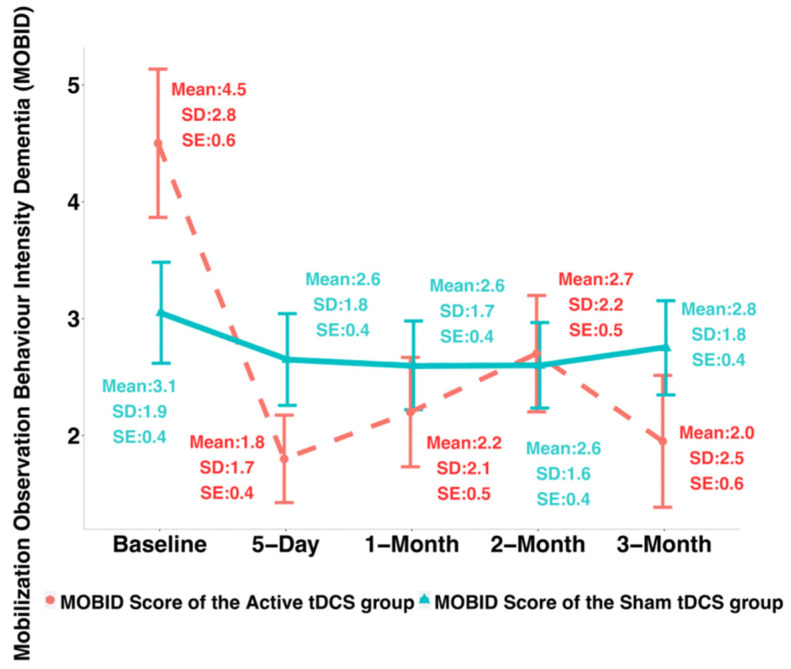
Mobilization Observation Behaviour Intensity Dementia (MOBID) scores over time of the active and the sham tDCS groups (*n* = 40). The dashed red line represents the mean MOBID scores of the active tDCS group, and the solid green line represents the mean MOBID score of the sham tDCS group.

To verify whether the improvement by tDCS can be sustained, we compared the clinical pain intensity changes of the active tDCS group from baseline to 3-month after intervention with the changes from baseline to 5 days using the paired Wilcoxon test for the treatment group. For both the NRS and the MOBID scores, no significant difference was found between 5-day changes and after-intervention 3-month changes. In addition, Table 3 and Table 4 (see also Figure 2 and Figure 3) present the results of profile analysis based on linear mixed-effects model, where the temporal trajectories of NRS and MOBID scores over time were compared between the active and the sham groups. The results showed that: (1) the temporal trend (i.e., slope) of NRS scores of the active tDCS group was not significantly different from that of the sham group (*p*-value = 0.22); (2) the profile of MOBID scores of the active tDCS group was significantly different from that of the sham group (*p*-value = 0.01).

We further employed the generalized estimating equation (GEE) model to examine the effects of selected covariates on NRS and MOBID scores over all the time points (Baseline, Day 5, Month 1, Month 2, and Month 3). The results showed that: (1) the group indicator (*p*-value < 0.001), age (*p*-value < 0.01), weight (*p*-value < 0.01), and race (Black African American vs. White, *p*-value < 0.01) were significantly associated with the changes of NRS over time; (2) the group indicator (*p*-value < 0.01), and age (*p*-value < 0.01) were significantly associated with the changes of MOBID over time. Note that the number of Black African Americans is only 1 such that we cannot draw any reliable conclusions regarding race differences. Finally, sex-stratified analysis was performed. There were no statistically significant differences found between males and females for both NRS and MOBID scores. For female participants, the NRS changes were found significantly different between the two groups (*p*-value < 0.01). For male participants, the NRS changes were found significantly different between the two groups (*p*-value = 0.02); also, weight was significantly associated with NRS changes (*p*-value < 0.01). The MOBID changes were different between the sham and the treatment groups (*p*-value < 0.01) for the male participants only; for the female group, both treatment (*p*-value < 0.01) and age (*p*-value < 0.01) were found significant with respect to MOBID changes.

#### Behavioral and Psychological Symptoms of Dementia (BPSD)

Changes related to BPSD from Baseline to day 5 were not statistically significant between the sham and the active tDCS groups (CMAI: Cohen’s d = 0.11, *p*-value = 0.95; NPI: Cohen’s d = 0.10, *p*-value = 0.92). Table 2 presents the BPSD score changes from baseline to day 5 and after-intervention month 3 of the two groups. For CMAI, the mean decrease was −2.35 ± 5.99 on day 5 from baseline for the active group and 2.95 ± 5.24 for the sham group; At 3-month after intervention, the mean decrease of CMAI was 5.30 ± 9.80 for the active group while only 3.90 ± 7.16 for the sham group. For NPI scores, the mean decreased by 1.55 ± 13.08 on day 5 from baseline for the active group, and it was 2.65 ± 8.39 for the sham group; at 3-month after intervention, the NPI scores decreased by 4.80 ± 9.36 for the active group, and the decrease was 2.05 ± 4.83 for the sham group. Therefore, CMAI and NPI were not considered in subsequent analyses.

### 3.2. Feasibility and Acceptability

A total of 40 ADRD patients were screened, and all of them met the inclusion criteria. All the 40 participants agreed to be randomly assigned, completed the full tDCS protocol, and attended the follow-up assessment. Participants expressed a positive perception of home-based tDCS and showed high levels of acceptability regarding their experience (as shown in Table 5), despite not being allowed to participate in other activities during the tDCS session. Overall, they found that the device was easy to use, agreed that the video conferences were helpful, felt confident using the device and that most people could learn how to use it quickly. Based on the Client Satisfaction Questionnaire (CSQ), the satisfaction rate of participants was high (CQS-8 = 28.65 ± 3.03) and no statistically significant difference was found between groups. Lastly, all participants tolerated home-based tDCS well without reporting any side effects or adverse events.

## 4. Discussion

This is the first randomized sham-controlled trial to examine the preliminary efficacy of home-based tDCS on clinical pain in older adults with early-stage ADRD. We showed that five 20-minute sessions of home-based tDCS were feasible and acceptable, and significantly reduced clinical pain intensity as measured by a NRS with a moderate effect size. Of note, this difference was clinically meaningful as illustrated by a reduction of 30% when compared to baseline [77], although the significant difference between groups was not maintained at 3 months. Clinical pain intensity as reflected by caregivers using the MOBID-2 scale was also significantly reduced after treatment in the active group when compared to the sham group with clinically relevant differences (i.e., a difference of at least 2 points on 0–10 NRS) [77] and a large effect size. Our findings illustrate a successful first step in planning for the use of this intervention in a larger scale study.

These results converge with our previous sham-controlled study using home-based tDCS with older adults suffering from chronic pain in which we obtained significant results on clinical pain intensity (as measured by NRS) [47]. These findings also support the appropriateness of our approach for the placement of tDCS in relation to brain regions to impact chronic pain in this population and are supported by tDCS guidelines for the treatment of pain [30]. Regarding feasibility and acceptability in patients with ADRD, the results are promising with no dropouts and no adverse events reported, and a high level of satisfaction with the home-based tDCS experience. This is also in line with prior numerous cognition-focused studies using tDCS and home-based tDCS among persons with ADRD [46,53,54,55,78]. Of note, the home-based approach is advantageous for older adults with chronic symptoms due to challenges with travel, especially in the setting of having chronic pain and cognitive deficits [79].

Our findings also suggest that the chronic pain experienced by individuals with ADRD involves central processing mechanisms since tDCS was able to impact pain intensity. Indeed, tDCS is a neuromodulation method potentially affecting pain processing pathways as shown in our studies using neuroimaging and conditioned pain modulation (CPM) measures [48,80,81,82]. We found significant changes in hemodynamic brain activity measured with functional near-infrared spectroscopy (fNIRS) [80,81,82], and simultaneously observed a significant increase in CPM in the active group as opposed to the sham group after tDCS [48], which coincides with tDCS studies using functional magnetic resonance imaging (fMRI) in both healthy subjects [83,84] and chronic pain patients [85,86]. In relation to ADRD, recent evidence suggests that there might be a link between pathological processes of ADRD and chronic pain [87]. Furthermore, an important study with a 27-year follow-up highlighted the differences between patients with and without dementia regarding their pain trajectories with pain intensity levels increasing closer to the time of dementia diagnosis, suggesting that pain is correlated with dementia and could be an early symptom of dementia [21].

There are a few limitations to consider. First, the assessment of pain intensity only included subjective measures: self-reporting and a proxy assessment provided by caregivers. However, this type of assessment by caregivers has shown to be a reliable approach for individuals with dementia [88,89] and is sensitive to pharmacological pain treatment protocols [63]. Additionally, our results with caregivers were in concordance with results obtained via the NRS with patients. Notably, caregivers’ post-treatment assessments could not be influenced by the participants’ answers as questionnaires were filled out in the laboratory and caregivers could not read the answers and vice versa. Monthly follow-up assessments done over the phone, however, could have been altered and digital data collection could be used to address this potential bias. Second, the significant difference between groups in pain intensity was not maintained at 3 months. This is explained by the fact that therapeutic effects of tDCS are known to subside. Indeed, our group and others observed this “wearing off” phenomenon although there are reports of tDCS displaying carryover effects, [35,39,47,90]. Lastly, we did not observe any significant changes on BPSD. However, these symptoms were mild creating a floor effect in addition to the short-term follow-up that did not allow for fluctuation of these symptoms over time. Moreover, studies using tDCS for dementia-related symptoms usually involve more tDCS sessions for a longer period [46,53,78].

Now that the feasibility, acceptability, and efficacy of home-based tDCS have been promising for pain relief in individuals with ADRD, a logical next step would be to increase the number of sessions to substantiate the effects of tDCS. Five sessions have been established to be the minimum number of sessions required to see improvements as repetition is key in tDCS approaches [30]. Given the numerous studies safely implementing an extensive number of sessions or an extended length of tDCS therapy for cognitive symptoms in ADRD [46,53,78] and findings from studies focused on dose–response effects suggesting that the number of responders to tDCS treatment may increase with ongoing treatment [56,91], tDCS maintenance therapy may be a promising avenue for long-term chronic pain relief in persons with ADRD. Maintenance tDCS for pain relief is still germinal but has been recently identified as a research priority in the field [92]. Interestingly, studies using tDCS for cognition and memory improvement in individuals with ADRD have observed a delayed progression or a stabilization of the condition when stimulation is used over a longer period of time up to 6 or 8 months [46,53], suggesting a potential role for tDCS as a maintenance therapy for ADRD itself. Furthermore, there should be more research on combining home-based tDCS with other nonpharmacological interventions that could optimize responsiveness of the brain to produce greater effects on chronic pain beyond what can be achieved with tDCS alone in those with ADRD. We have obtained promising results by combining home-based tDCS with mindfulness-based meditation in older adults with knee osteoarthritis [39]. Lastly, using neuroimaging data could complement our understanding of the effects of tDCS on pain in this population [30,80].

## 5. Conclusions

We have demonstrated that home-based and remotely supervised tDCS can reduce pain intensity in persons with ADRD and that this approach is feasible and acceptable. These findings warrant the need for further studies with larger samples investigating various doses of tDCS and their long-term effects on chronic pain.

## Figures and Tables

**Table 1:** Baseline Demographic and Clinical Characteristics of the Participants.

	Sham tDCS (N = 20)	Active tDCS (N = 20)	*p* Value
Age, years, M (SD)	71.90 (9.20)	74.15 (6.32)	0.37
Gender, *n* (%)			0.72
Male	5 (25.0%)	6 (30.0%)	
Female	15 (75.0%)	14 (70.0%)	
Race, *n* (%)			0.55
Asian	1 (5.0%)	1 (5.0%)	
Black African	0 (0.0%)	1 (5.0%)	
White	19 (95.0%)	17 (85.0%)	
Hispanic or Latino	0 (0.0%)	1 (5.0%)	
BMI, kg/m^2^, M (SD)	26.92 (5.35)	26.73 (5.35)	0.91
Marital Status			0.64
Married	11 (55.0%)	12 (60.0%)	
Widowed	3 (15.0%)	2 (10.0%)	
Divorced	4 (20.0%)	4 (20.0%)	
Separated	1 (5.0%)	0 (0.0%)	
Never married	0 (0.0%)	1 (5.0%)	
Living with partner	0 (0.0%)	1 (5.0%)	
Refused	1 (5.0%)	0 (0.0%)	
Education, *n* (%)			0.73
Some school but did not complete high school	1 (5.0%)	0 (0.0%)	
High school degree	4 (20.0%)	5 (25.0%)	
Two-year college degree	3 (15.0%)	2 (10.0%)	
Four-year college degree	3 (15.0%)	6 (30.0%)	
Master’s degree	4 (20.0%)	4 (20.0%)	
Doctoral degree	5 (25.0%)	3 (15.0%)	
NRS, M (SD)	37.95 (30.83)	45.75 (27.54)	0.27
MOBID-2, M (SD)	3.05 (1.93)	4.50 (2.84)	0.07
CMAI, M (SD)	36.80 (10.87)	37.80 (9.06)	0.51
NPI, M (SD)	9.25 (12.17)	12.85 (15.65)	0.42

Note. tDCS = transcranial direct current stimulation, M = Mean, SD = Standard Deviation, BMI = Body Mass Index; NRS = Numeric Rating Scale of Pain, ranging from 0 (no pain) to 100 (worst pain imaginable); MOBID-2, Mobilization-Observation-Behavior-Intensity-Dementia, ranging from 0 to 10, with higher scores indicating more pain behaviors; CMAI: Cohen Mansfield Agitation Inventory, ranging from 29 to 203, with higher scores indicating more agitation behaviors; NPI (Q.1–12): Neuropsychiatric Inventory, ranging from 0 to 144, with higher scores indicating greater symptoms. Wilcoxon rank-sum test was used for continuous variables, and Chi-squared test was used for categorical variables.

**Table 2:** Comparison between groups on changes from baseline in clinical pain (NRS, MOBID) and other symptoms (CMAI, NPI).

Variable	Sham tDCS (N = 20)	Active tDCS (N = 20)	Effect Size (d)	*p*-Value
NRS changes between 5-day and baseline	−3.30 (14.81)	−13.55 (14.90)	0.69	0.02
NRS changes between 3-month after intervention and baseline	−8.30 (27.59)	−18.7 (27.48)	0.39	0.23
MOBID changes between 5-day and baseline	−0.40 (1.67)	−2.70 (2.39)	1.12	<0.01
MOBID changes between 3-month after intervention and baseline	−0.30 (1.81)	−2.55 (3.46)	0.82	0.06
CMAI changes between 5-day and baseline	−2.95 (5.24)	−2.35 (5.99)	0.11	0.95
CMAI changes between 3-month after intervention and baseline	−3.90 (7.16)	−5.30 (9.80)	0.16	0.53
NPI changes between 5-day and baseline	−2.30 (11.59)	−2.9 (15.08)	0.10	0.57
NPI changes between 3-month after intervention and baseline	−1.00 (5.88)	−7.05 (11.96)	0.05	0.09

Note: Mean (Standard Deviation) are presented in the first two columns; NRS = Numeric Rating Scale; MOBID: Mobilization Observation Behavior Intensity Dementia Pain Scale; CMAI: Cohen Mansfield Agitation Inventory; NPI: Neuropsychiatric Inventory. Wilcoxon rank sum test was used for NRS, CMAI, and NPI score changes and *t* test was used for MOBID score changes.

**Table 3:** Profile analysis results for Pain Numeric Rating Scale (NRS).

Variable	Estimate	Standard Error	95% Confidence Intervals		T Value	*p* Value
Intercept	37.95	5.74	26.84	49.06	6.61	0.50
Day 5	−3.30	4.88	−12.67	6.07	−0.68	0.50
Month1	−3.30	4.88	−12.67	6.07	−0.68	0.31
Month2	−4.95	4.88	−14.32	4.42	−1.01	0.09
Month3	−8.30	4.88	−12.67	6.07	−1.70	0.34
Active tDCS	7.80	8.12	−17.67	1.07	0.96	0.34
Day 5: Active tDCS	−10.25	6.90	−23.50	3.00	−1.49	0.14
Month1: Active tDCS	−14.80	6.90	−28.05	−1.54	−2.15	0.03
Month2: Active tDCS	−16.15	6.90	−29.40	−2.89	−2.34	0.02
Month3: Active tDCS	−10.40	6.90	−23.65	2.85	−1.51	0.13

**Table 4:** Profile analysis results for Mobilization Observation Behaviour Intensity Dementia (MOBID).

Variable	Estimate	Standard Error	95% Confidence Intervals		T Value	*p* Value
Intercept	37.95	5.74	26.83	49.06	6.61	<0.01
Day 5	−3.30	4.88	−12.67	6.07	−0.68	0.50
Month1	−3.30	4.88	−12.67	6.07	−0.68	0.50
Month2	−4.95	4.88	−14.32	4.21	−1.02	0.31
Month3	−8.30	4.88	−17.67	1.07	−1.70	0.09
Active tDCS	7.80	8.12	−7.91	23.51	0.96	0.34
Day 5: Active tDCS	−10.25	6.90	−23.50	3.00	−1.49	0.14
Month1: Active tDCS	−14.80	6.90	−28.05	−1.55	−2.15	0.03
Month2: Active tDCS	−16.15	6.90	−29.40	−2.89	−2.34	0.02
Month3: Active tDCS	−10.4	6.90	−23.65	2.85	−1.51	0.13

**Table 5:** Descriptive results for the tDCS experience questionnaire (*n* = 40).

Items of the tDCS Experience Questionnaire	Mean (SD)
1. It was easy to prepare the device and accessories (0–10)	8.98 (2.36)
2. The device was unnecessarily complex (0–10)	0.80 (2.26)
3. The device was easy to use (0–10)	9.45 (1.80)
4. I felt the video conferences with a technical person were helpful (0–10)	9.27 (1.94)
5. I would imagine that most people would learn to use this device quickly (0–10)	9.60 (0.98)
6. The device was cumbersome to use (0–10)	1.25 (2.68)
7. I felt confident using the device (0–10)	9.35 (1.85)
8. I needed to learn a lot of things before I could get going with this device (0–10)	2.20 (3.62)
9. The effectiveness of the treatment increased over the course of treatment (0–10)	6.90 (3.48)
10. Overall, I felt that transcranial electrical stimulation treatment benefited me (0–10)	6.80 (3.44)

Note: one score was collected per participant. Questionnaire was completed by patient and/or caregiver depending on who manipulated equipment.

## Data Availability

Not applicable.

## Supplementary Materials

The following supporting information can be downloaded at: https://www.mdpi.com/article/10.3390/jcm12020401/s1, Figure S1: CONSORT Flow Diagram; Table S1: Multivariable regression results for Numerical Rating Scale (NRS) and Mobilization Observation Behaviour Intensity Dementia (MOBID) score changes between day 5 and baseline.
