# Supplementary material for: Feasibility, Acceptability, and Efficacy of Home-Based Transcranial Direct Current Stimulation on Pain in Older Adults with Alzheimer’s Disease and Related Dementias: A Randomized Sham-Controlled Pilot Clinical Trial
Source: J Clin Med. 2023 Jan 4;12(2):401. doi: 10.3390/jcm12020401 (PMC9860690; doi:10.3390/jcm12020401)

Table S1. Multivariable regression results for Numerical Rating Scale (NRS) and Mobilization Observation Behaviour Intensity Dementia (MOBID) score changes between day 5 and baseline.

Results for NRS

Parameter	Estimate	Standard Error	p-value
Intercept	-26.74	24.19	0.23
Active tDCS	10.80	4.91	0.047
Age	0.19	0.33	0.57
Gender	-1.31	5.7	0.81
Race			
Asian	7.13	11.12	0.53
Black African American	-24.89	15.97	0.13
Hispanic or Latino	14.49	15.54	0.36

Results for MOBID

Parameter	Estimate	Standard Error	p-value
Intercept	-5.259	3.13	0.10
Active tDCS	2.14	0.64	<0.01
Age	0.04	0.04	0.34
Gender	-0.41	0.74	0.58
Race			
Asian	-2.00	1.44	0.17
Black African American	-5.17	2.07	0.02
Hispanic or Latino	0.58	2.01	0.78

CONSORT
TRANSPARENT REPORTING of TRIALS

Figure S1. CONSORT Flow Diagram

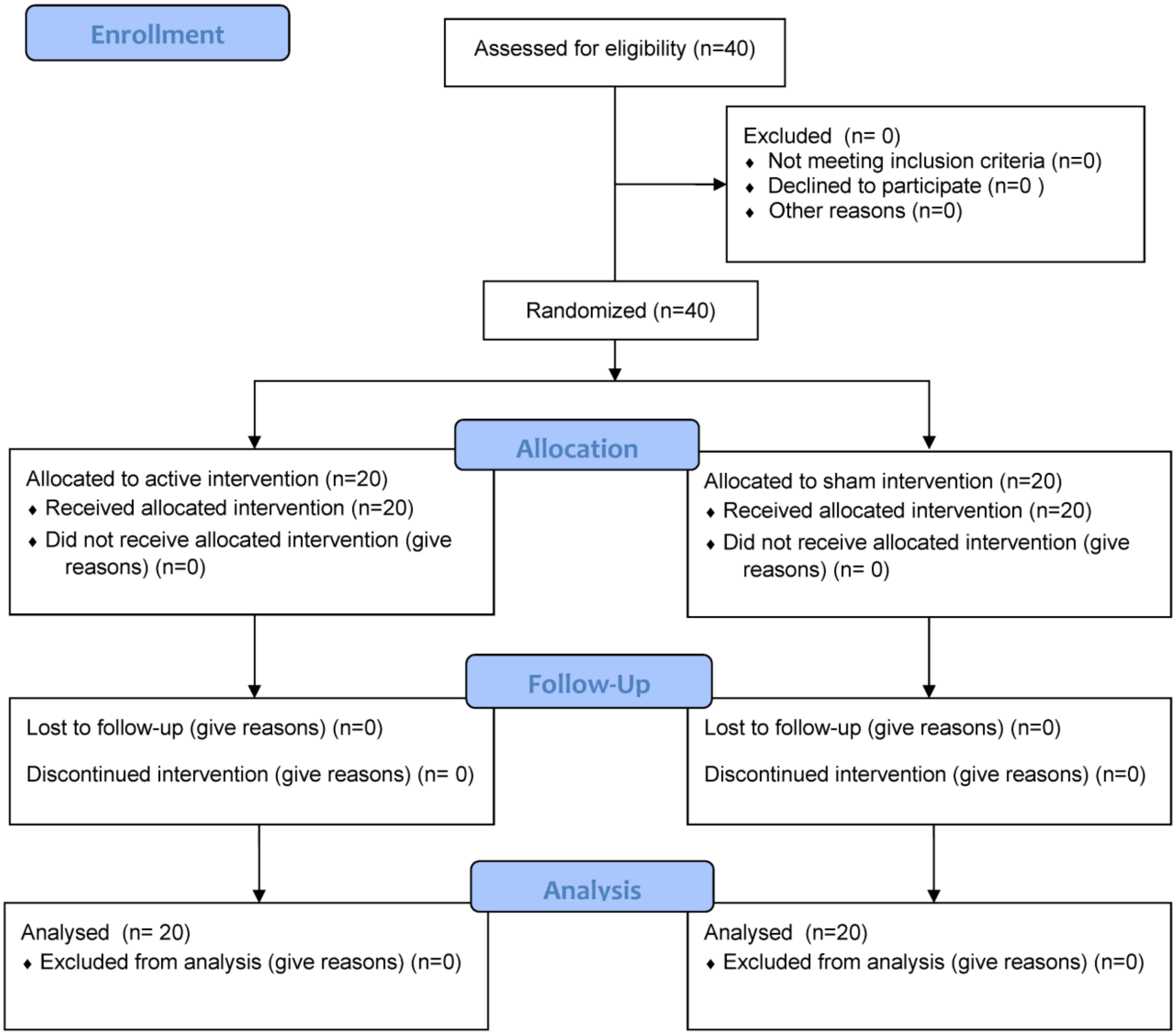

Supplement: Supplementary file 1 [file jcm-12-00401-s001.zip › jcm-2082836-supplementary.pdf]
